# Brain Stimulants

**Published:** 1873-09

**Authors:** 


### BRAIN STIMULANTS.

A prominent clergyman in a neighboring city writes us, that for many years he has been in the habit of limiting his use of tea and coffee, and his "occasional cigar," to the latter part of the week, and, as he fancies, with the result of being able to compose with less effort than when he has either abstained entirely from their use, or when, as once or twice, he has indulged in them continuously for a brief period.

Herein is a valuable suggestion to brain workers in any profession the exigencies of which call for occasionally increased and severe efforts. Tea, coffee, tobacco and alcohol, by retarding the changes in the tissues in the body, which is their physiological action, are supposed to allow the energy thus conserved to manifest itself in the higher form of cerebral activity—in simpler language, they are stimulants to the nervous system ; and, in the proper dose, there can be no question that they do exalt and stimulate brain action. But there is equally no question that the retarded tissue changes at the expense of vitality generally—the vitality of the body, that is, its health and strength, being ever in relation to the newness of the atoms which compose the body—and these tissue-changes, the work of waste and repair, must be accelerated in some manner, and to a corresponding extent, in order to preserve the balance.

The obvious lesson to be gained from these facts is, that during periods of intense and unusual mental activity—a lawyer in trying an engrossing case, a banker during a financial stress, a company officer at periods of increased responsibility, an editor or political leader carrying through an important measure—that at such times brain-work may be done with more facility and at less expense, by a judicious use of this class of agents. Provided, however—provided that the balance be struck at once when the neccessity for them is obviated.

The means of restoring the balance include first, abstinence from the agents themselves ; second, comparative rest for the brain ; and lastly, and quite as important as the preceding, those measures which accelerate tissue changes, and of which the essential ones are physical exercise and bathing— notably the Turkish bath—and nutritious, easily assimilated food, by the first of which, the breaking down of the older particles, and the excretion of poisonous waste-matter are facilitated, and so tissue change in the interest of waste is promoted, while the last furnishes material for renewal and growth.

With such a regimen, based on an intelligent application of means to ends, we would have fewer cases of men prematurely breaking down under efforts they might make with ease, did they only know how and when to open the throttle-valve, or to put on the brakes. This view of the subject must not be construed into argument for a mere sensual indulgence. It is intended for men as they are, and with regard to conditions as they exist. These agents are used, and probably always will be. They have their uses ; and knowledge of them will do more to prevent their abuse than the wholesale condemnation which frequently arises from ignorance.—*Hygiene.*

